# A Tumor-Selective Monoclonal Antibody from Immunization with a Tumor-Associated Mucin Glycopeptide

**DOI:** 10.1038/s41598-019-42076-2

**Published:** 2019-04-05

**Authors:** Kevin R. Trabbic, Kaitlyn Whalen, Karin Abarca-Heideman, Li Xia, J. Sebastian Temme, Elijah F. Edmondson, Jeffrey C. Gildersleeve, Joseph J. Barchi

**Affiliations:** 1 Chemical Biology Laboratory, Center for Cancer Research, National Cancer Institute, Frederick, MD, USA; 2 Rockland Immunochemicals Inc., Limerick, PA 19464 USA; 3 Pathology and Histotechnology Lab, Leidos Biomedical Research Inc., Frederick, MD USA

## Abstract

We have previously studied the generation of immune responses after vaccination with tumor-associated carbohydrate antigen (TACA)-containing glycopeptides from the tandem repeat (TR) sequence of MUC4, an aberrantly expressed mucin in pancreatic adenocarcinomas. A specific lead antigen from that study containing the Thomsen-Friedenreich TACA disaccharide facilitated the pursuit of a monoclonal antibody to this synthetic hapten. Initial evaluation of polyclonal antiserum resulting from immunization with a KLH conjugate of this glycopeptide into rabbits showed high titer antibodies by ELISA assays, and selective immunoreactivity with MUC4^+^ cells by western blot and flow cytometry techniques. Glycan microarray analysis showed an intriguing binding pattern where the antiserum showed near complete specificity for MUC4 TR glycopeptides and peptides, relative to all components on the array. Tissue staining also showed distinct tumor specificity to pancreatic tumor tissue in relation to normal pancreatic tissue, with a preference for more aggressive tumor foci. Based on this data, we produced a monoclonal antibody whose binding and reactivity profile was similar to that of the polyclonal serum, with the added benefit of being more specific for the N-terminal glycosylated peptide domain. This epitope represents a novel immunogen to potentially develop diagnostic antibodies or immunotherapies against various MUC4-positive cancers.

## Introduction

Pancreatic cancer (PC) is the fourth leading cause of death in the United States. Despite only approximately 50,000 cases a year, pancreatic cancers patients have the lowest 5 year survival rate of only 6%^1^. It is has been predicted that a surge in cases by 2030 could advance pancreatic cancer to become the second leading cause of cancer deaths in the US^2^. The abysmal 5-year survival rate is primarily due to the very late stages in which the disease is diagnosed. PC is often asymptomatic until a stage where the primary tumor has already metastasized to other organs, rendering therapeutic intervention largely unsuccessful. Survival will only improve with better methods of early detection through the identification and validation of biomarkers that report on disease staging and aggressiveness. Novel tumor-specific biomarkers can also be targets of therapeutic intervention, either by small molecules or biologics (antibodies, immune therapy).

At present, there are very few biomarkers for the most common form of PC, pancreatic ductal adenocarcinomas (PDAC). In addition, therapeutic options for both early and advanced PDAC are limited to only a handful of small molecule chemotherapeutics that have not changed for several years. In fact, a recent review by Goel and Sun opens with the statement “Despite a few breakthroughs in therapy for advanced disease in the recent years, pancreatic ductal adenocarcinoma continues to remain one of the most challenging human malignancies to treat^3^.” Notwithstanding, there are a variety of new therapeutic options that are in various stages of clinical evaluation. Several of these are forms of immunotherapy, either vaccines or antibodies, that target a range of PDAC surface markers. Vaccine preparations such as GVAX^4^, IMM-101^5^ and Algenpantucel-L^6^ have all been evaluated in different stages of clinical trials; however, none have emerged as viable therapeutic options as most trials in combination with various small molecules failed to reach statistically significant endpoints (see for example ascopost.com and reference therein)^7,8^.

One family of highly overexpressed and aberrant proteins on many tumor cells are mucins^9^. These are a group of large membrane-bound or secreted glycoproteins that serve as a physical barrier to protect epithelial cells from damage and to help maintain a chemically-balanced physiological environment^9,10^. Their overall structures are varied, but they primarily exist as two domains with one large extracellular subunit and a smaller, transmembrane cytosolic domain. A high percentage of the extracellular domain consists of tandem repeat (TR) motifs of ~16–22 amino acids that contain a large degree of serine (Ser), threonine (Thr), and proline (Pro) residues. Many of the Ser and Thr residues exist in clusters and are O-glycosylated^9^. Upon neoplastic transformation, both the expression of the proteins and composition of the covalently linked oligosaccharides are modified to tumor-associated forms. Tumor-associated mucin glycans are truncated from larger branched structures to units with only one, two and three sugars. Three of the most prevalent of these so-called tumor associated carbohydrate antigens (TACAs) are, (1) the Tn antigen (GalNAcα-*O*-Ser/Thr), (2) the TF antigen (TF_ag_, Galβ1-3GalNAcα-*O*-Ser/Thr), and (3) the sialyl-Tn (Neu5Acα2-6-GalNAcα-*O*-Ser/Thr) antigen^11,12^. These shorter glycan chains serve to expose underlying peptide sequences that were masked by the larger “normal” oligosaccharides, and these newly presented glycopeptide epitopes are the subject of immune surveillance (hence the term “antigen”). The more robust antibody response to these antigens leads to a better prognosis and less aggressive tumors^13,14^. Consequently, these TACAs, as well as the associated mucin protein sequences, have been the focus of numerous studies for immunotherapeutic intervention against various cancers bearing aberrant mucin carbohydrates^11,15–22^. The most widely studied cancer–associated mucin is MUC1, which is overexpressed in a variety of cancers, especially breast and prostate cancers^23–30^. Another of the membrane bound mucins, MUC4, is sparse or absent on normal pancreas tissue but aberrantly expressed in PC/PDAC, making MUC4 a marker for both pre-cancerous and PC lesions^31^. However, antitumor therapeutic intervention targeting MUC4 has not been as widely pursued^32^ as that of MUC1^10,15,25,26,28,29,33–35^.

**Figure 1:**
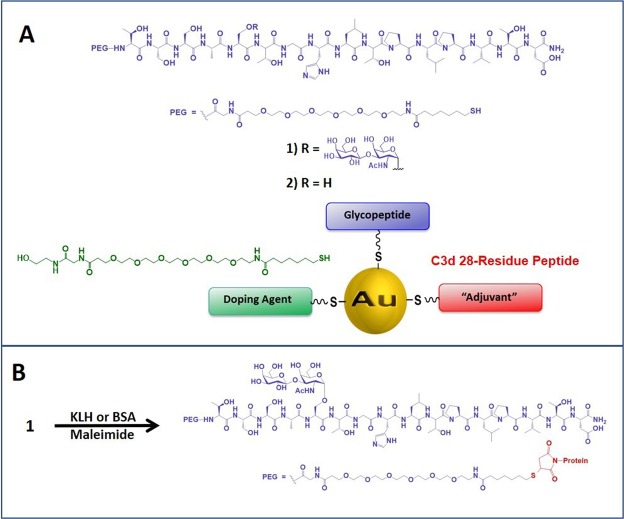
Antigen/vaccine structures and conjugation procedures used in this work. (**A**) structure of the 16-residue tandem repeat peptide from MUC4 with a TF_ag_ at position Ser5 (5TF_ag_-TR_MUC4_) and the PEG linker used to conjugate to gold nanoparticles to construct our vaccine platform (shown), as well as to prepare KLH and BSA conjugates (**B**).

Our lab has been studying the immunogenicity and therapeutic design of agents based on MUC4 TR glycopeptides bearing the TF TACA (TF_ag_). The TF_ag_ is a pan-carcinoma structure rarely found on normal epithelial cells and is a marker for tumor aggressiveness and metastasis^36,37^. We have previously developed a gold nanoparticle (AuNP) vaccine construct that can elicit an immune response in mice to specific conjugates on the gold surface after intraperitoneal administration with no adjuvant^38^. During that work, a specific glycopeptide construct we prepared where the TF_ag_ was attached to the fifth serine residue from the N-terminus of the TR peptide (5TF_ag_-TR_MUC4_, Fig. 1) was shown to be a lead candidate for development based on several desirable features of this molecule. Initial evaluation by glycan microarray analysis of murine polyclonal serum obtained commercially from a KLH conjugate of this synthetically linked-hapten showed that the serum only bound to our MUC4 TR glycopeptides displayed on the array (unpublished results, *vide infra*). No other TF_ag_-containing molecule interacted with the serum, suggesting a defined specificity for the epitope from polyclonal serum. The selectivity of the serum prompted us to pursue the development of a monoclonal antibody to this glycopeptide structure. This report describes the production and preliminary evaluation of affinity purified polyclonal serum and a novel mAb generated from a series of hybridomas. We show through various assays (Western blots, ELISA, Flow Cytometry, tissue staining and glycan microarray analysis) that this mAb is tumor selective and has preferential binding to a glycosylated peptide structure relative to the peptide sequence alone.

## Results

The selection of the 5TF_ag_-TR_MUC4_ construct stemmed from results of our initial studies showing that vaccination of mice with a tumor-associated glycopeptide, a C3d adjuvant peptide and a doping agent coated on small gold nanoparticles can generate an immune response to the glycopeptide^38^. The TF_ag_ was chemically ligated to various positions on the TR sequence. Immunization with the construct with the TF_ag_ in the Ser5 position (compound **1**, Fig. 1A) produced high titer IgG antibodies, and mice that were implanted with 4T1 breast tumors had longer survival than those vaccinated with other TR-glycopeptides where the TF_ag_ disaccharide was linked to other positions (unpublished data). Murine polyclonal serum was therefore raised to the antigen **1** conjugated to KLH (Fig. 1B) and evaluated on a diverse glycan microarray,^39–41^ which contained all of the glycopeptides that were used in the original vaccine study and at least 30 other molecules that contained the TF_ag_ in various forms. The serum was highly specific for the MUC4 TR glycopeptides and showed no binding to any other TF_ag_-containing array component (Supporting Information Fig. S1). Based on the remarkable specificity displayed in these preliminary results, a second batch of polyclonal serum was raised in rabbits in a collaboration with Rockland Immunochemicals (Limerick, PA) and part of this serum was affinity purified on a Sepharose column conjugated with the thiol-terminated molecule **1** as described in *Materials and Methods*. This affinity-purified serum was evaluated in several assays, where selectivity and tumor-specificity were assessed. In the first sections below, we describe the results for the rabbit polyclonal serum. In the following sections, we will then describe the development and characterization of a mouse monoclonal antibody that was produced based on the data obtained with the polyclonal antibodies.

### Results for rabbit polyclonal antiserum

#### Western Blots

Several pancreatic cell lines are available where mRNA levels of MUC4 have been determined, allowing for comparisons of interactions between immune serum with cells with low and high MUC4 expression^42^. The serum bound strongly to proteins in cell lysates from MUC4^+^ PDAC cells such as HPAC and HPAF but not to proteins in cell lysates from MUC4^−^ PDAC cells such as PANC-1 (Fig. S2) or to other MUC4^−^ tumor cells such as T24 bladder carcinoma cells (data not shown). The bands show a varied pattern for each of the MUC4^+^ cell lines; these differences are most likely caused by a small amount of protein degradation or a binding to the various (differentially glycosylated) tumor isoforms that are usually present when MUC4 is expressed on the cell surface. However, the patterns were similar for both the pAb serum and mAb we derived from that mixture (*vide infra*).

#### Evaluation of epitope structure

**Figure 2:**
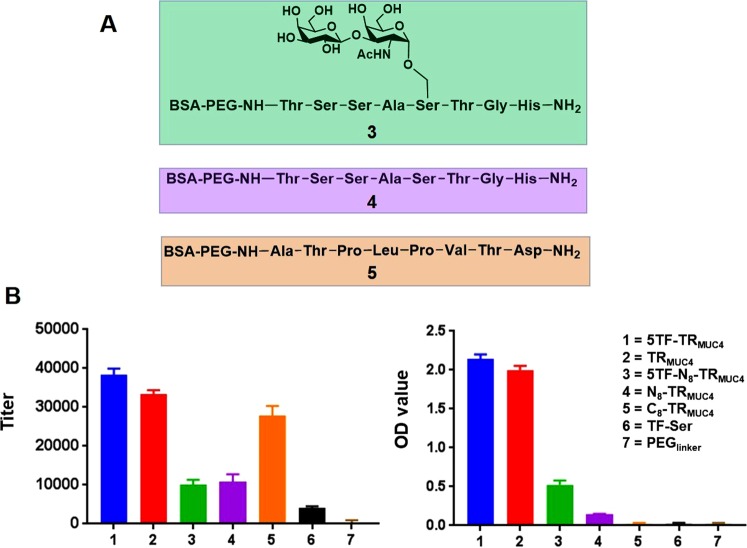
**(A**) Structures of the 8-mer TR “halves” (compounds **3**–**5**) for epitope mapping. (**B**) ELISA IgG titers for AP-polyclonal serum (left) and OD values for mAb binding (right) to the various constructs described in the inset.

The glycan array technology we used to screen the initial mouse antiserum (*vide infra*) employs BSA conjugated neoglycoproteins as array components^41^. Thus, we prepared BSA conjugates of our TR glycopeptides to employ as both (1) array components and (2) reagents for ELISA and other binding assays. BSA conjugates of 5TF_ag_-TR_MUC4_ and the unglycosylated TR peptide sequence (Compound **2**, Fig. 1) were prepared by thiol conjugation to maleimide-BSA (Fig. 1B). In addition, we also prepared a simple TF_ag_-disaccharide-BSA conjugate along with a BSA-conjugated PEG linker without the peptide and terminated by a hydroxyl group, similar to what we used as a control in our vaccine study^38^. These reagents allowed the stratification of the various moieties on the antigen that are recognized by the polyclonal antiserum. As can be seen in Fig. 2, both the glycopeptide and unglycosylated peptide interact strongly with the antiserum with a slight preference (1.2:1) for the glycosylated antigen. The serum titers were much more concentrated for IgG than IgM antibodies (Fig. S3). Very weak binding was observed to the simple TF_ag_ disaccharide, suggesting the importance of the peptide sequence and possibly its preferred conformation for binding the antiserum. We were very pleased to discover that binding of the serum to the linker alone was minimal to almost absent. This was a welcomed result as many linkers that are based on coupling chemistry such as triazoles from click chemistry reagents can elicit strong immune responses and dampen the response to the desired antigen^43,44^.

In order to focus in on the actual features of the glycopeptide that are recognized by the affinity-purified antiserum, we also synthesized two unglycosylated 8-mer “halves” of the TR peptide (N_8_-TR_MUC4_ and C_8_-TR_MUC4_) along with the N-terminal 8-mer containing a TF_ag_ at the Ser5 position (5TF_ag_-N_8_-TR_MUC4_, Fig. 2A). Each of these three peptides were attached to the linker described above and conjugated to BSA (See Supporting Information for experimental procedures). ELISA data showed that the serum bound primarily to the C-terminal half of the TR (Fig. 2B). Binding to both the N-terminal 8-mer peptide and glycopeptide was similar, but about 2.5-fold weaker than to the C-terminal 8-mer (Fig. 2B). This data suggests that the sugar may serve to redirect the response to the unglycosylated part of the sequence, at least within the context of the polyclonal immune serum.

#### Evaluation of Whole Cell Binding

**Figure 3:**
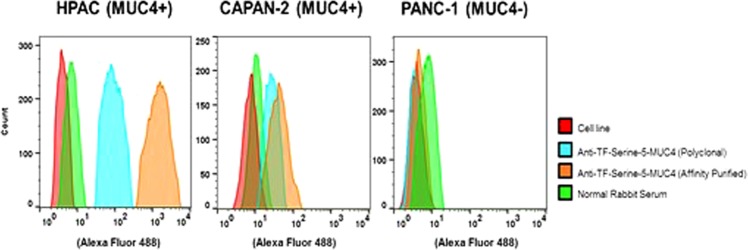
Flow cytometry histograms of pAb binding to MUC4^+^ and MUC4^−^ cell lines.

**Figure 4:**
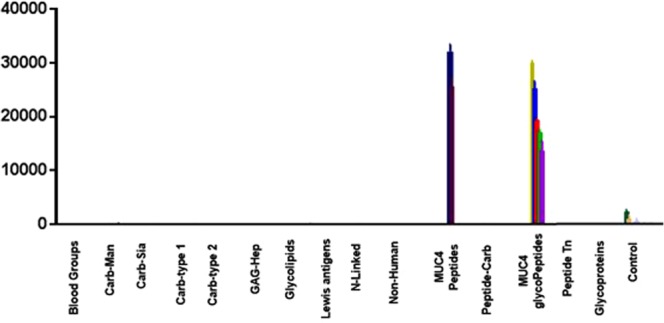
Plot of signal intensity relative to glycan families for pAb (1:1000 dilution) binding the glycan microarray. Only the MUC4 peptides/glycopeptides that printed bound to the serum, while no other TF-containing molecule bound.

We used flow cytometry to determine the cellular specificity of serum binding. Both MUC4^−^ and MUC4^+^ cell lines were employed in these experiments as defined in the *Materials and Methods* section. HPAC and CAPAN-2 (MUC4^+^) cells and a MUC4^−^ cell line, PANC-1, were used and the data are shown in Fig. 3. As is shown by the fluorescence intensity plots, the MUC4^+^ cells strongly interact with the serum where there is little to no effect with MUC4^−^ cell lines, confirming the selectivity for those cells expressing MUC4. Interestingly, a stronger interaction was seen with the HPAC cell line compared to CAPAN-2 cells, suggesting a higher expression of MUC4 in the HPAC line (Fig. 4A,B). This result “mildly contradicts” the reported greater quantities of MUC4 mRNA in CAPAN-2 cells (++++) relative to HPAC cells (+++)^42^, however, mRNA levels may not correspond directly with antibody binding to cells since this measurement may not translate protein levels. In addition, the mean fluorescent intensities (MFI) from both 5TF_ag_-TR_MUC4_ antibodies (serum and affinity purified) were shown to have significant fluorescent intensity over unimmunized rabbit serum. This selective recognition further confirms that the serum raised can recognize tumor cells expressing a valid marker for aggressive pancreatic tumors.

#### Glycan Microarray Analysis

A multi-component glycan microarray was used to characterize the specificity of the generated antiodies. The microarray consisted of >700 carbohydrates, oligosaccharides, glycoaminoacids and glycopeptides that represent a range of glycans from various cellular presentation (*N*-liked, *O*-linked, glycolipids, gangliosides)^39^. The affinity purified anti-serum was used (at both 1:200 and 1:1000 dilutions) to minimize any potential cross reactivity with off target materials in the polyclonal mixture. The array data are briefly summarized in Fig. 4 where the array components are grouped by molecular family. As discussed above, the glycan array format utilizes BSA conjugates as array components. The results recapitulate what we observed earlier with the polyclonal mouse serum: A remarkable selectivity for only the MUC4 peptide/glycopeptides was seen whereas *no other array component bound the serum with any appreciable affinity*. The array contained the four glycopeptides we used for our previous vaccine study along with the unglycosylated peptide and the linker alone. As shown in the ELISA data (*vide supra*), no binding was observed to the linker itself. The selectivity for the MUC4 constructs on the array was again very high; at a dilution of 1:1000, the signals for the MUC4 glycopeptides are at least 100-fold higher than any other glycan or peptide on the array.

These data confirm that the affinity-purified polyclonal antibody is highly specific for MUC4 peptides and glycopeptides derived from the synthetic hapten used for immunization.

#### Immunohistochemical Staining

**Figure 5:**
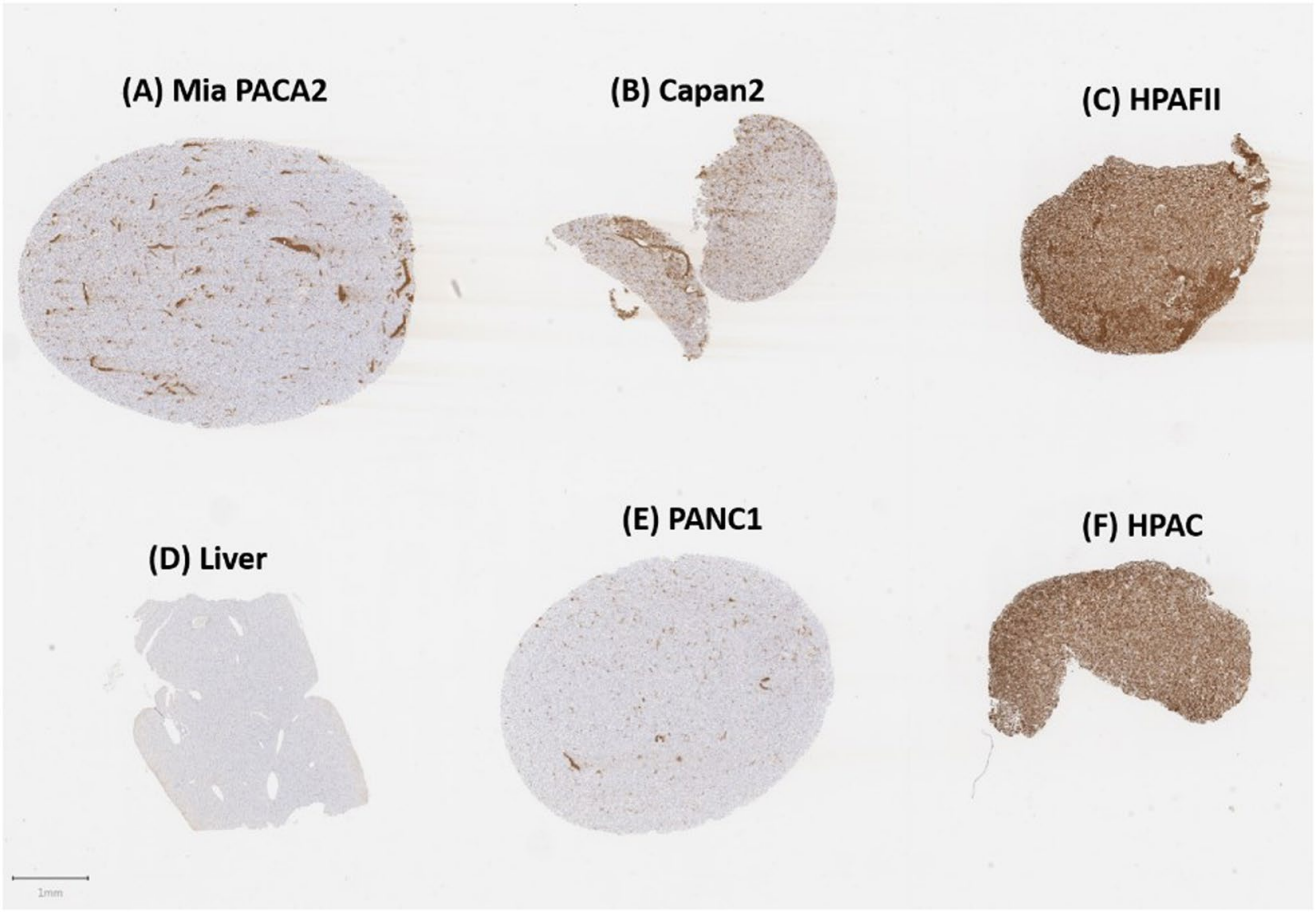
Staining of various MUC4^+^ and MUC4^−^ cell pellets with affinity-purified pAb serum. Some non-specific, acellular staining is seen within MiaPACA2 (MUC4^−^) cells. No staining was observed for control liver and PANC1 (MUC4^−^) cell pellets while HPAFII and HPAC pellets that are known to express large amounts of MUC4 were strongly and diffusely positive.

We initially analyzed both MUC4^+^ and MUC4^−^ cells for staining by the affinity purified serum. Cells were disrupted according to the procedures in *Materials and Methods* and cell pellets were fixed and stained. As seen in Fig. 5, there is defined differential staining of the cell pellets in MUC4^+^ (HPAF-II, HPAC) and MUC4^−^ (Mia PaCa-2, PANC-1, negative control) cells.

Figure 5C, F show the strong positive staining for HPAF-II and HPAC cell pellets relative to the negative controls (liver cells, Fig. 5D and PANC-1 PDACs, Fig. 5E). Mild positive staining (~5% of cells, Fig. 5B) was seen in the CAPAN-2 cell line, also deemed MUC4^+^ by others in the literature. There is some non-specific staining that can be attributed to recognition by the anti-serum in the extracellular space (Fig. 5A,D,E). This can be seen in the Mia PaCa-2 cell line (Fig. 5B) in which cells were interpreted to be negative, but extracellular spaces within the sample contained positive staining.

**Figure 6:**
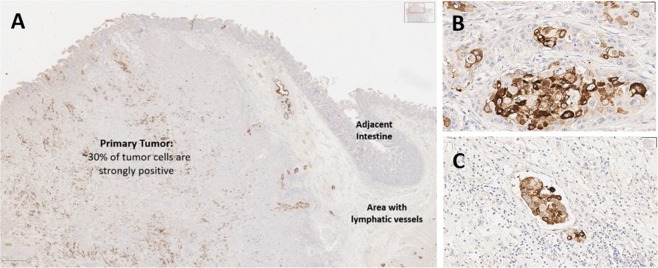
A section of pancreatic adenocarcinoma with vascular invasion is evaluated for MUC4 expression. The primary tumor mass (**A**) is heterogenous for MUC4 expression, with some neoplastic cells demonstrating strong positive staining while others are negative. At higher power, MUC4 staining is intense within some neoplastic cells (**B**) and not expressed in others. Numerous tumor emboli are present within lymphatic vessels in this tissue section; essentially all neoplastic cells invading vascular structures are positive for MUC4, suggesting the antiserum recognizes tumor clones more likely to produce metastasis within the heterogenous neoplastic tissue.

A slide containing pancreatic adenocarcinoma acted as a positive control to comprehensively probe the amount of staining and the specific cellular compartments that were immuno-reactive. Figure 6A,B show that approximately 30% of cells within the primary tumor were strongly positive for cytoplasmic staining with the anti-serum, while a small number of cells were also positive in the adjacent normal tissue, including intestinal crypts. This is not surprising due to the high expression of MUC4 in the gastrointestinal tract^23^. Interestingly, we also found that tissue adjacent to the primary tumor (including the tunica submucosa of the intestine) contained nests of strongly positive neoplastic cells observed within these endothelial lined structures (interpreted to be lymphatics due to lack of RBCs), consistent with vascular invasion (Fig. 6C). Essentially all (~100%) of the neoplastic cells in these endothelial lined structures are positive for MUC4, suggesting that the antiserum bound a higher percentage of aggressive tumor cells displaying vascular invasion than the more heterogenous staining observed in the primary tumor (Fig. 6B).

**Figure 7:**
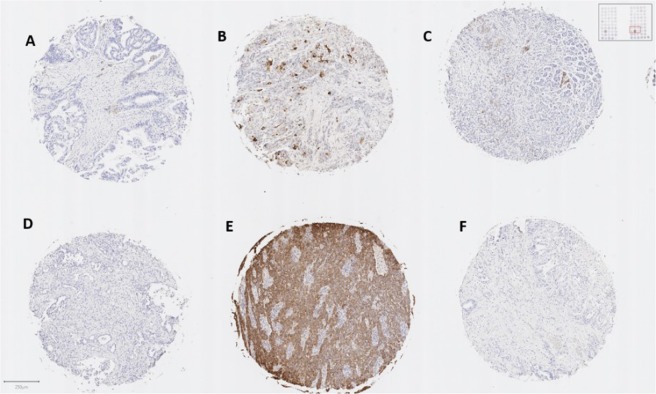
Tissue microarray staining revealed no staining (**D**,**F**), mild and multifocal cytoplasmic staining (**A**,**C**), moderate and multifocal cytoplasmic staining (**B**), or intense and diffuse positive staining (**E**).

A tissue microarray (TMA) containing a set of 100 clinically assessed samples of pancreatic tissue from patients (80 PDAC tumors, 20 normal) was also screened with the serum for possible staining of TF/MUC4 expressing cellular components. Representative staining patterns for the PDACs printed on this array are shown in Fig. 7. While most tumors were essentially negative (Fig. 7C,D,F), many tumor cores showed variably staining, which could be divided into four categories: (1) mild to moderate staining in cytoplasmic compartments (Fig. 7A), (2) multifocal, moderate to strong cytoplasmic expression (Fig. 7B), (3) strong and diffuse staining (Fig. 7E) or (4) no staining. Image analysis was performed to quantify staining, represented by a pseudo-color mask as blue (negative) to red (strongly positive) with green representing stromal tissue (Fig. S4). Of the 80 tumor tissues, 13 were positive in the +2 to +3, with 7 rating a +3, while at least another 10 were considered mildly (+1) positive. It is important to point out that all 20 normal tissue samples were negative (See Supporting Information Excel file #1 for a list and description of all tissue on the TMA).

### Monoclonal antibody (mAb) production and analysis

Several hybridomas were produced through standard techniques as outlined in the *Material and Methods* section. We evaluated 10 cellular supernatants with varying selectivities for either the peptide, glycopeptide or both. We chose a specific clone (F5, Table S1) that had a slight preference for the glycopeptide for production and purification. We performed similar assays as run for the polyclonal serum and showed that the mAb data recapitulated all the results obtained above for the polyclonal serum. MUC4^+^ cell lines interact much more than those that do not express MUC4 in Western blot (Fig. S2), ELISA (See below) and flow cytometry experiments (Fig. S5). An almost equal interaction is seen between the mAb and the HPAF-II cell line as seen in the data for the affinity purified polyclonal serum, whereas little to no interaction is seen with MiaPaca2 cells (MUC4^−^).

### Evaluation of monoclonal antibody selectivity

**Figure 8:**
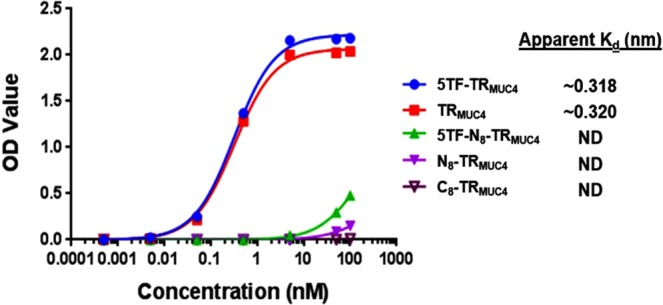
Concentration vs. OD curves from ELISA data on F5 for K_d_ determination. Calculated apparent K_d_’s are shown.

We used ELISA and glycan microarrays to assess the selectivity of antibody F5. Similar binding patterns were seen with F5 as with the polyclonal antiserum, with some intriguing and distinct differences. The mAb had similar binding to the 5TF_ag_-TR_MUC4_ glycopeptide and the 16-mer unglycosylated structure, both in ELISA and glycan array assays (Fig. 2 and Supporting Information Excel file #2). We were able to calculate apparent K_d_ values for the mAb binding to both the 5TF_ag_-TR_MUC4_ and TR_MUC4_ constructs to be nearly equal affinity in the sub-nanomolar range (~320 pM, Fig. 8). The most interesting result was the effect on binding of the 8-mer “halves” described above. While the pAb serum was selective for the non-glycan-containing C-terminal half, the F5 prefers the glycosylated N-terminal half (Figs 2 and 8). Although it was not possible to calculate apparent K_d_ values for the antigen “halves”, extrapolation would yield values at least 3 orders of magnitude greater (~150–250 nM). Hence when choosing clones that were potentially selective for the glycopeptide, we fortuitously chose a hybridoma that produced a mAb that altered its preference for the glycan portion of the epitope. This was partially corroborated by the glycan array data which was run at 6 dilutions (Supporting Information Excel file #2). Only MUC4 glycopeptides bound on the array as described above for the polyclonal serum, with both the peptide and 5TF_ag_-TR_MUC4_ glycopeptide binding almost equally well. In addition, although weaker binding was observed to the N-terminal constructs overall, the glycosylated N-terminal 8-mer bound ~2–2.5 fold higher than the unglycosyated N-terminal peptide. The mAb had zero binding to the C-terminal 8-mer. In addition, adding a TF-disaccharide to the 6 position (5, 6 Di-TF) maintained binding while adding one to the threonine at position 10 (in the C-terminal end; 5, 10 Di-TF) reduced binding relative to the 5, 6 Di-TF. A construct with a single TF at position 6 (6-TF) is detrimental to binding. Thus, our antigen structures and immunization protocol tend toward production of immunoglobulins that maintain a modicum of the antigen glycopeptide selectivity after mAb production, even though only one disaccharide was attached to the backbone.

### F5 mAb Immunohistochemistry

**Figure 9:**
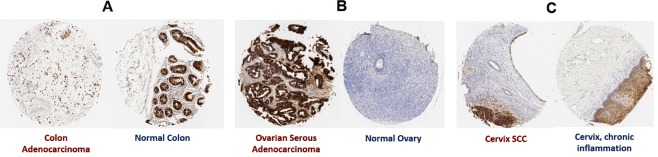
Tissue array staining of tumor and normal tissue was evaluated on multiple histotypes and (**A**) moderately stains colonic adenocarcinoma cells as well as adjacent normal colon tissue; (**B**) stains ovarian serous adenocarcinoma (but not adjacent normal ovarian tissue) and (**C**) moderately stains cervical squamous cell carcinoma as well as the squamous epithelium from chronically inflamed cervical tissue.

Cellular TMA staining was repeated for F5 and the result were similar to those obtained with the polyclonal serum, although there was slightly less non-specific staining with the monoclonal antibody relative to the polyclonal serum (Fig. S6). In addition to the PDAC/control staining, we employed F5 to examine staining of a tissue microarray comprised of a variety of solid tumor types. Normal and malignant tissues from esophagus, stomach, colon, prostate, liver, lung, kidney, breast, bladder, lymph node, skin, pancreas, testis, tongue, and placenta were evaluated. Neoplasms arising from colonic epithelium, cervical epithelium, and ovarian serous epithelial cells were positive. Normal colon and cervix were also positive for MUC4, whereas normal ovary samples (which do not contain fallopian tube, the likely site of origin for ovarian serous papillary adenocarcinoma) were negative. It is known that normal gastrointestinal tissue, including colon^45,46^ and cervix^47,48^, can express relatively high amounts MUC4 (see for example, https://www.proteinatlas.org/ENSG00000145113-MUC4/tissue). These data are summarized in Fig. 9.

## Discussion

The near ubiquitous presence of MUC4 in PDAC to the exclusion of normal pancreatic tissue, along with the successful targeting of MUC4-based signaling in anticancer drug discovery suggests that this protein is a valid biomarker for therapeutic development. We have been interested in this protein for these reasons, as well as the reports from several groups that suggest the presence of the TF_ag_ on tumor-associated MUC4 tandem repeat regions (prostate^49^ and pancreatic tumors^50^). The choice of the 5TF_ag_-TR_MUC4_ construct described here stems from our original work with a MUC4 vaccine construct^38^ and other evaluations of MUC4-derived TF_ag_-containing glycopeptides (unpublished work). We wanted to further establish this glycopeptide as a solid lead by developing an antibody-based tool/probe to identify the presence of motifs that maintain the conformational features of 5TF_ag_-TR_MUC4_ in pancreatic tumors. Immunization of rabbits with a 5TF_ag_-TR_MUC4_-KLH conjugate produced selective IgG and IgM antibody responses (since rabbits only produce a single IgG immunoglobulin, isotyping was not required). There was a small (~1.2) increase in recognition for both the IgG and IgM repertoire towards the 5TF_ag_-TR_MUC4_ glycopeptide compared to the free (unglycosylated) peptide. Notwithstanding, we showed by several techniques that our affinity-purified serum binds MUC4-containing tumor cell lysates by Western blotting and MUC4-containing tumor cells by flow cytometry. Importantly, the MUC4 TR-peptide/glycopeptide epitope sequence was nearly exclusively recognized on a glycan microarray, while having minimal to no binding to all other TF_ag_-containing molecules on the array (see Supporting Information Excel file #2 for a list of array components). Since BSA, KLH and the PEG linker we used to attach the glycopeptide to KLH were also printed on this microarray, we were able to quickly show that there was no cross reactivity of the antibodies with any other linker/carrier molecules. Even though binding to carrier proteins (BSA, KLH, tetanus toxin) is a time-honored method to coax many weakly immunogenic epitopes (such as carbohydrates) to generate immune responses, there are drawbacks to this technology due to the many highly immunogenic epitopes also present on these complex macromolecules. Thus, conjugates with these proteins can redirect the immune response to other epitopes of the carrier—or the linker that attaches the carrier to the epitope. We were thus very pleased to see minimal antibody recognition to the flexible thiol-PEG linker (Figs 1 and 2). These results bode well for using the molecular entities of our construction for future vaccine development.

Several factors may contribute to why the Abs only slightly favor the glycosylated TR over the unglycosylated peptide: (1) We have only used one TF_ag_ in this constructs and its effect on conformation could be small, (2) the presence of a glycan helps populate a peptide conformation that is recognized by the immune system that does not contain the glycan atoms or (3) the presence of the glycan redirects the immune response to the C-terminal end of the peptide which is the motif recognized by the immune system. To this end, we also prepared the two terminal halves of the peptide, conjugated them to BSA and performed ELISA experiments to more closely map the epitope that is recognized by the polyclonal and monoclonal entities. We were pleased to find that while the polyclonal serum preferred the C-terminal non-glycosylated half of the peptide, evolution to a monoclonal species resulted in selection of specific clones where the preference tended more toward the N-terminal glycosylated half of the epitope. This result is not completely surprising, since the pAb was cross-adsorbed to non-glycosylated peptide, so antibodies that did cross react with that part of the antigens were removed from the pool. This method should thus enrich for those antibodies with specificity for the presentation displayed by the glycosylated peptide. Although overall much lower than the full peptide sequence, the mAb binding was now higher compared to the non-glycosylated N-terminal peptide or the C-terminal peptide. In addition, a 5, 6 Di-TF antigen construct also bound whereas glycosylation in the C-terminal half was detrimental to binding. The enrichment of immunoglobulins for this part of the epitope was welcomed as our aim was to generate glycopeptide-specificity. The results reported here are certainly not conclusive in that regard, but signify the beginning of our efforts in this area.

Whichever, if any, feature described above is in play, the structure of our immunogen is one that seems to be highly tumor-selective as we see very minimal staining of normal tissue as evidenced by tissue staining with both purified polyclonal serum and the monoclonal antibody. While not all PDAC samples on the slides were positive, there were no normal samples that stained with our Abs. We progressed to a series of several other solid tumor samples along with matched normal tissue and found strong positive staining for cervical and colon tumors, results that enhance the utility of this mAb. Some normal staining was also shown with these tissue, so the selectivity for these tumor types is not as high as with the PDAC/normal pancreas samples. Most intriguing was that the tumor tissue that was stained by mAb F5 seemed to tend toward those that were more aggressive. A plot of the pathological H-scores in relation to tumor grade is shown in Supporting Information Fig. S7. Of the neoplasms evaluated, significantly increased MUC4 expression was observed in histologically high-grade tumors (grade 3); thus, assays to determine MUC4 expression may be a useful prognostic indicator.

We now have access to both polyclonal and monoclonal antibodies to this target, which offers some advantages^51^. The pAbs can recognize diverse epitopes on the target protein, which makes them suitable for a variety of analysis methods such as western blots and IHC, while mAb’s have a high degree of homogeneity and specific epitope detection making them more fit for quantification experiments and therapeutic applications. The results reported here stand in contrast to a commercial antibody (8G7) that was raised to a similar although unglycosylated peptide hapten^52^. This antibody binds equally well to normal and tumor tissue. The peptide used for 8G7 immunization was also a 16-mer, but frame shifted by 4 amino acids relative to our 16-mer TR sequence. In addition, there are three variations in amino acid residues in the 8G7 epitope sequence. This may account for some of the differences in specificity we see between our polyclonal/monoclonal antibodies and 8G7. We thus feel our antibody will be a significant complement to 8G7 and expand the applications of this family of monoclonal antibodies. Our work is continuing with other antigens based on the MUC4-TR sequence where multiple residues are modified with TACA glycans and will be reported in due course.

## Materials and Methods

### Western Blots

To 1.0 × 10^6^ cells in 190 μL of PBS was added 10 μL of 25X RIPA buffer. After vortexing, 75 μL of 4X LDS sample buffer (previously combined with 20 μL of 0.5 M DTT) was added. The mixture was centrifuged, the supernatant was isolated and protein concentration was estimated by UV spectroscopy on a Thermo NanoDrop spectrophotometer. This cellular lysate (30 µg) was analyzed by SDS-PAGE (2 h elution at 100 V). The eluted proteins were transferred from the gel to a polyvinylidene difluoride (PVDF) membrane and blocked with 4% BSA for 2 h. Primary anti-serum or monoclonal antibody were incubated on the membrane at 37 °C for 2 h and washed several times. Secondary horseradish peroxidase-conjugated antibodies were incubated with the membrane for 1 h, washed and detected by UniGlow chemiluminescent substrate (Rockland Immunochemicals). Western blots were imaged using an ImageQuant LAS 4000.

### Antigen conjugation and antibody production

Synthetic peptide was coupled to keyhole limpet hemocyanin (KLH) as the carrier protein using maleimidobenzoyl‐N‐hydroxysuccinimide ester (MBS) crosslinkers. For monoclonal antibody development Balb/c mice and for polyclonal antibody development New Zealand White rabbits were used. Animals were given an initial immunization with Complete Freund’s Adjuvant (CFA) and subsequent booster injections with Incomplete Freund’s Adjuvant (IFA) to elicit and maintain antigen recognition by the host animal via an ID/SC protocol. Monoclonal antibodies were screened for clones that showed reactivity to target, combined with no cross reactivity to non-MUC4 peptides and subsequently purified by Protein A chromatography. A complete list of selected clones is shown in Table S1 (F5 PN: 200-301-GY2, Rockland Immunochemicals Inc.) Polyclonal antibodies were affinity purified against target and cross adsorbed to remove non MUC-background reactivity. Polyclonal antibody PN 600-401-GY2. Monoclonal and polyclonal antibodies were further characterized as described in the main text. Specific aspects of the characterization of the conjugation, immunizations, and purification of the polyclonal antibodies are confidential standard operating protocols of Rockland Inc. Animal studies were performed at Rockland Immunochemical’s fully owned subsidiary Oxford Care Facilities, Schwenksville, PA. All animal related work was approved by Rockland Immunochemicals Inc. Institutional Animal Care and Use Committee (IACUC) and performed with authorization and certification for animal use (NIH Assurance #A4062-01; USDA License #R 23-R-0184).

### Cell Culture

All cells were grown at 37 °C. HPAC cells were cultured in DMEM:Ham’s F12 (containing 5% FBS, 0.002 mg/mL of insulin, 0.005 mg/mL transferrin, 40 ng/mL hydrocortisone, and 10 ng/mL of epidermal growth factor); PANC-1 and MIA PaCA-2 were cultured in 10% MEM; CAPAN-2 was cultured in 10% FBS in McCoys 5 A medium and HPAF-II and CCD 841 CoN were cultured in 10% EMEM.

### Flow Cytometry

The general flow cytometry procedure was as follows: 1.0 × 10^6^ cells were incubated with a 1:50 dilution of the polyclonal antiserum, 1:50 dilution of normal rabbit serum or 50 ug/mL of affinity pure polyclonal antiserum for 1 hr on ice and in the dark. The cells were washed three times in 3% BSA in PBS pH 7.3. Cells were fluorescently labeled using goat anti-rabbit IgG Alexa Fluor 488 at 1:500 dilution and incubated at 4 °C for 30 min. The cells were washed three times and fixed with 1% PFA. Cells were injected on a BD FACSCalibur II instrument and analyzed with FlowJo (https://www.flowjo.com/).

### ELISA Data

BSA constructs (0.3 ug/mL) were coated onto Immunlon 4BX plates and screened using the following antisera anti-5TF_ag_-TR_MUC4_ serum (1:300), affinity pure anti-5TF_ag_-TR_MUC4_ polyclonal eluant (15 µg/mL) and unimmunized rabbit serum (1:300). The plates were blocked for 1 h with 3% BSA and washed three times with PBS with 0.005% Tween 20. The primary antiserum was added to the well plates at the aforementioned concentrations and serially diluted by half Log10 dilutions and incubated at 37 °C. After 2 h, the plates were washed, and secondary antibodies (anti-IgG and anti-IgM) labeled with alkaline phosphatase were used to detect bound rabbit primary antibodies. The serum was diluted 1:1000 and incubated for 1 h at 37 °C with the appropriate reagent and the plates were washed 3x. PNPP (10 mg) in pH 9.8 DEA buffer was added to the wells and incubated for 30 min. The optical density was read at 405 nm.

### Glycan microarray fabrication

Detailed experimental procedures for the production of neoglycoproteins, the fabrication of the microarray, and the assay and data analysis have been reported previously^53,54^. Briefly, glycans or glycopeptides were chemically conjugated to either bovine serum albumin (BSA, A3059, Sigma-Aldrich, St. Louis, MO) or human serum albumin (HSA, A8763, Sigma-Aldrich, St. Louis, MO) to produce neoglycoproteins to be printed on the microarray. The average number of glycans conjugated per molecule of albumin was determined by MALDI-TOF MS (AXIMA Confidence reflectron, Shimadzu Biotech, Kyoto, Japan) and is indicated by the number following the abbreviation of the array component names. Full details of the preparation and characterization of the neoglycoproteins has been previously published^55^.

For the microarrays used in this experiment, a total of 738 array components were printed in duplicate on epoxide-coated slides (SuperEpoxy2, ArrayIt, Sunnyvale, CA) using a NanoPrint™ 2 LM60-2 arrayer (ArrayIt, Sunnyvale, CA). Print buffer for each component was composed of 2.5% glycerol, phosphate buffered saline (PBS), 0.0005% triton X-100, and 0.005 µg/mL Atto 532 (a soluble and washable print dye used to evaluate the integrity of the printed spots). Six SMP2 microspotting pins (ArrayIt, Sunnyvale, CA) in a 3 × 2 printhead arrangement were used for this print. Each slide contains 8 identical arrays in a 1 × 8 format. Humidity level was maintained at ~60% in the arraying chamber to minimize evaporation during the print. The printing spot size was ~80 μm. Following print completion, printed slides were vacuum sealed and stored at −20 °C until use in binding assay experiments. Quality of the printed slides was evaluated by scanning several slides using an InnoScan 1100 AL Fluorescence Scanner (Innopsys; Chicago, IL) to detect printing defects (for a representative image, see Supplementary Fig. S8, A). A representative slide from each print batch was then tested against a set of lectins (ConA, WGA and HPA) and mouse anti-blood group A and anti-blood Group B. Values from the quality control slide were compared against expected values from previously validated arrays.

### Glycan microarray binding assays

The array slide was warmed to room temperature and scanned to determine print integrity. After being mounted with an 8-well slide module (Grace bio-labs, Bend,OR), the 8 identical and separate arrays were blocked with 400 µL of 3% w/v BSA PBS buffer for 12 hours at 4 °C. The microarray was rinsed with 3 × 400 µL of phosphate buffered saline with 0.05% tween-20 (PBST). Diluted mouse mAb (100 µL) or 100 µL anti-MUC4 polyclonal serum (Rockland, Limerick, PA) in 3% w/v BSA PBST buffer was added to the washed microarray and incubated at 37 °C with gentle shaking (100 rpm) for 2 hours. The microarray was rinsed 4 times with PBST and washed 2 × 2 minutes with PBST. Following washing, 200 µL of fluorescently labeled secondary antibodies (Cy3-labeled anti-mouse antibody for monoclonal Ab or Cy3-labeled anti-rabbit antibody for polyclonal, 1:500 in 1% BSA w/v in PBST, Jackson ImmunoResearch Laboratories, West Grove, PA) was added to the microarray and incubated at 37 °C with gentle shaking for 1 hour. The microarray slide was extensively washed, removed from the slide module, and dried by centrifugation (5 min at 200 rcf, Eppendorf 5810 R, Hauppauge, NY). The microarray slide was scanned at 5 μm resolution and 35 linespeed.

### Glycan microarray data analysis

Images were analyzed with GenePix Pro 7.0 software (Molecular Devices Corporation, Sunnyvale, CA). GAL files specific to the array number were aligned with slide component. Print defects (e.g. missing spots from pre-assay scan) were flagged and excluded from analysis. The final intensity value for an antibody/serum-neoglycoprotein interaction was calculated from the average of duplicate spots in each well. For representative array images, see Supplementary Fig. S8 (**B** rabbit polyconal serum; **C**, murine mAb F5). Full glycan microarray data can be found in the Supporting Excel file.

### Tissue sections, tissue arrays, and cell pellets

To evaluate expression of MUC4 in tissue sections, formalin-fixed, paraffin embedded (FFPE) tissue microarray (TMA) slides containing normal and malignant pancreatic tissues (U.S. Biomax, Inc., PA1002a) were submitted for immunohistochemistry staining along with a control slide containing a large section of pancreatic adenocarcinoma and adjacent normal tissue. Finally, cell pellets with characterized MUC4 expression were collected, fixed, and paraffin embedded within the same block to validate MUC4 IHC sensitivity and specificity. Briefly, FFPE cell pellet slides were created as follows: Approximately 10 million cells were collected from flasks without enzymatic treatment and centrifuged; thrombin stock was added to the pellet and mixed briefly and placed on ice. Fibrinogen stock was next added and incubated for 3 minutes at room temperature. The resulting pellet of clotted cells was collected and fixed in 10% neutral buffered formalin at room temperature. Pellets were then embedded in paraffin and routinely processed as tissue sections.

### Immunohistochemistry

All FFPE materials, including tissue section, TMA, and cell pellet slides, were stained with the same methods, with anti-MUC4 antibody (Rockland 600-401-GY2, Rabbit, 1:1000). IHC staining was performed on Leica Biosystems’ BondMax autostainer with the following conditions: Heat-induced epitope retrieval 1 (Citrate) for 20 minutes, the primary antibody (1:1000), and the Bond Polymer Refine Detection Kit (LeicaBiosystems #DS9800). Isotype control reagents were used in place of the primary antibody for negative controls.

### Pathology

Slides were digitized with an Aperio ScanScope scanner (Leica) at 200X in a single z-plane. Tissue microarrays containing normal and malignant pancreatic tissues, FFPE cell pellets, and pancreatic adenocarcinoma with adjacent normal tissue were quantified for MUC4 expression using image analysis. A detection classifier was built using annotation examples of normal exocrine pancreas, pancreatic neoplasia, and stroma tissues to discriminate tumor and normal pancreatic cells from adjacent stromal cells using a random trees algorithm. Cell detections, annotation examples for machine learning, and cell type classification were supervised by a board-certified veterinary pathologist (EFE). IHC staining was quantified using thresholds validated with known positive controls and reported as an H-score for each sample^56^.

## Supplementary information

Supporting Info PDF

Supplementary Dataset 1

Supplementary Dataset 2

Supplementary Dataset 3

## Data Availability

All materials are freely available to those who request procedures and/or synthetic materials. The antibodies are licensed to Rockland Immunochemicals and will be available (for a fee) after publication of this work at their website at https://rockland-inc.com/Default.aspx.
