# Supplementary material for: A Tumor-Selective Monoclonal Antibody from Immunization with a Tumor-Associated Mucin Glycopeptide
Source: Sci Rep. 2019 Apr 5;9:5662. doi: 10.1038/s41598-019-42076-2 (PMC6450958; doi:10.1038/s41598-019-42076-2)
Supplement: Supplementary file 1 — Supporting Info PDF [file 41598_2019_42076_MOESM1_ESM.pdf]

Supporting Information

A Tumor- Selective Monoclonal Antibody from Immunization with a Tumor-Associated Mucin Glycopeptide

Kevin Trabbic, Kaitlyn Whalen, Karin Abarca-Heideman, Xia Li, J. Sebastian Temme, Elijah Edmonson, Jefferey C. Gildersleeve and Joseph J. Barchi Jr.*

Figure S1……………………………………………………….Page S2

Figure S2……………………………………………………… Page S2

Figure S3……………………………………………………… Page S3

Figure S4……………………………………………………… Page S3

Figure S5……………………………………………………… Page S4

Figure S6……………………………………………………… Page S4

Figure S7……………………………………………………….Page S5

Table S1……………………………………………………… Page S5

Figure S8……………………………………………………….Page S6

BSA Conjugates……………..…………………………………….Page S7

Scheme S1……………………………………………………...Page S7

MALDI data for BSA Conjugates…………………………………...Pages S8-S10

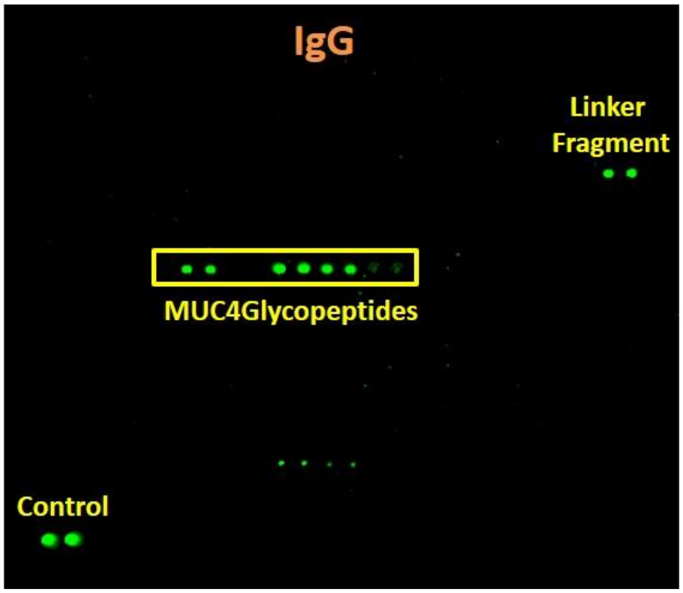

Figure S1. Original array scan of mouse polyclonal sera raised to $5TF_{ag}$-TR_{MUC4}. Only MUC4 peptides/gycopeptides bound (330 array components); the linker fragment also bound the sera.

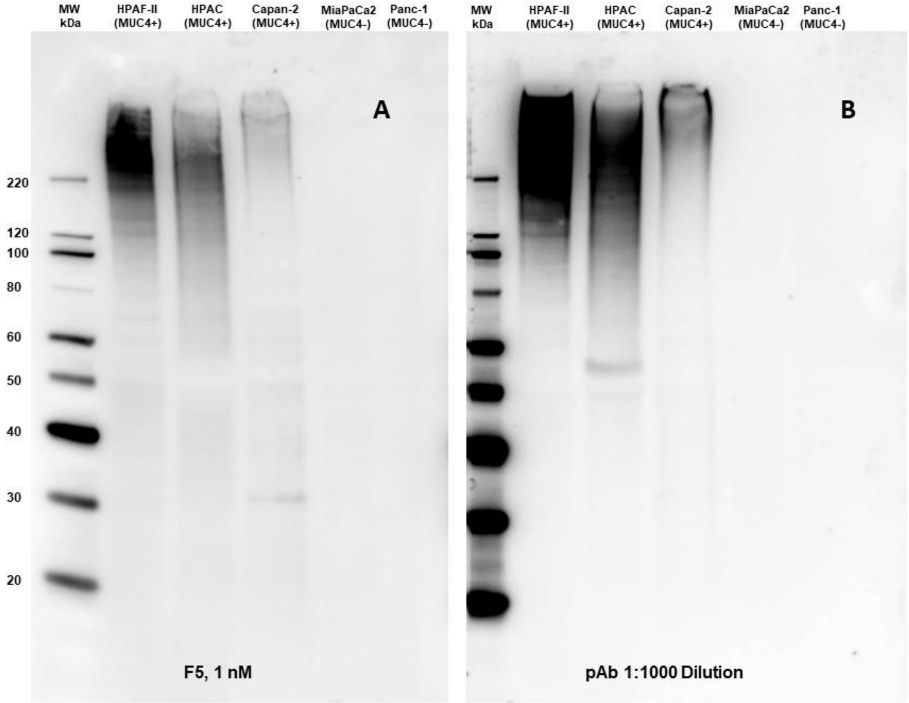

Figure S2. Western blot of affinity-purified polyclonal sera shows binding to $MUC4^+$ but not to $MUC4^-$ pancreatic tumor cells. (A) Blots of the mAb F5 at 1 nM concentration. (B) Blots of the pAb at 1:1000 dilution.

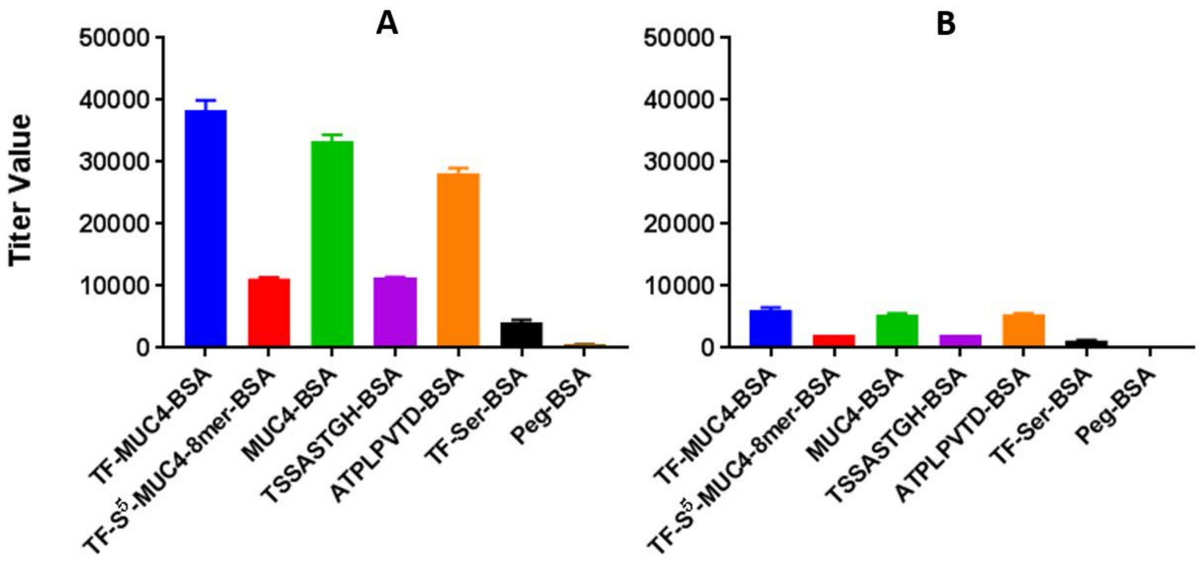

Figure S3. Comparison of antibody isotype titers for AP-polyclonal serum. (A) IgG values and (B) IgM values.

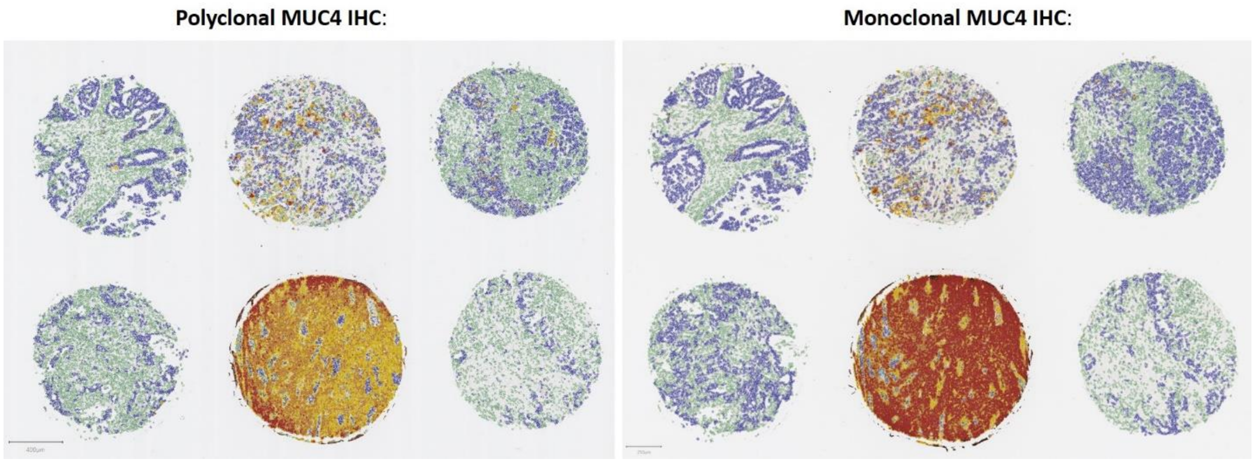

Figure S4. Immunohistochemical staining of tissue array with both AP-polyclonal sera (left) and the monoclonal antibody (right) against pancreatic tissue through digital pathology; tissue cores are visualized using a pseudo-color image mask analysis as follows: Green = Stroma; Blue = negative; Yellow = +; Orange = ++ and Red = +++.

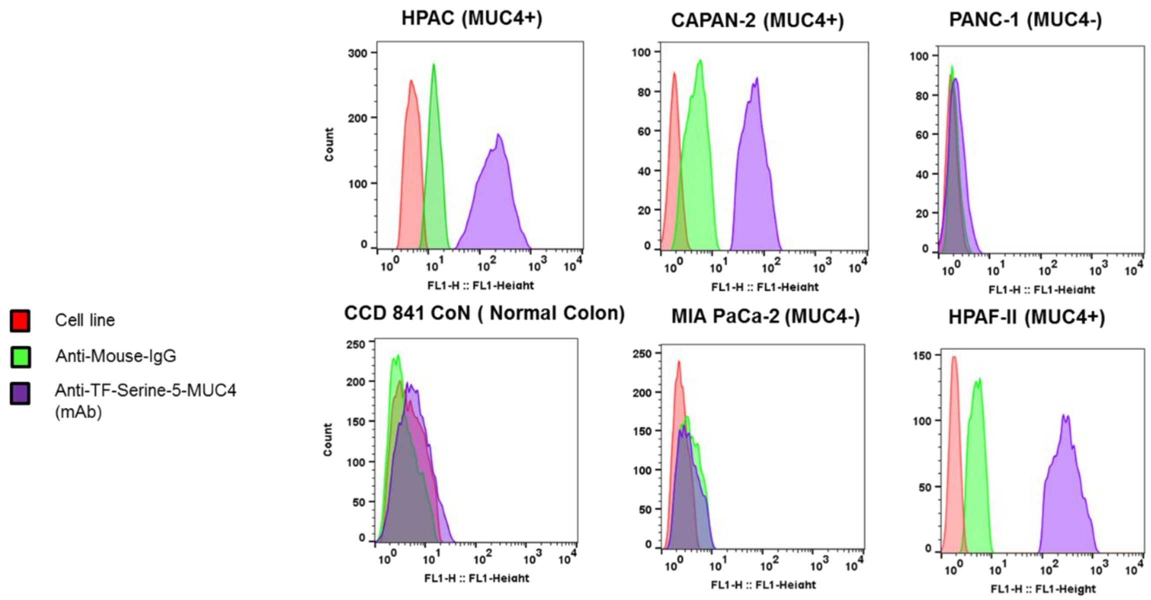

Figure S5. FACS analysis of the monoclonal antibody against both MUC4$^+$ and MUC4$^-$ cell lines.

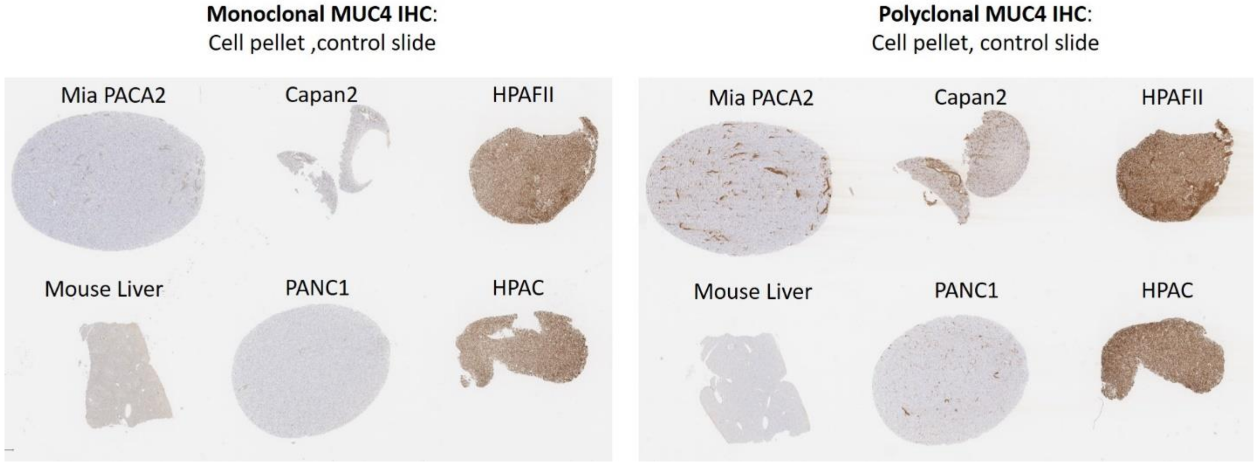

Figure S6. Comparison of monoclonal and polyclonal immunohistochemical staining in cell pellets of known positivity and mouse liver tissue. Both monoclonal and polyclonal anti-MUC4 produces expected specific staining in positive cell lines; however, polyclonal reagents have increased non-specific staining.

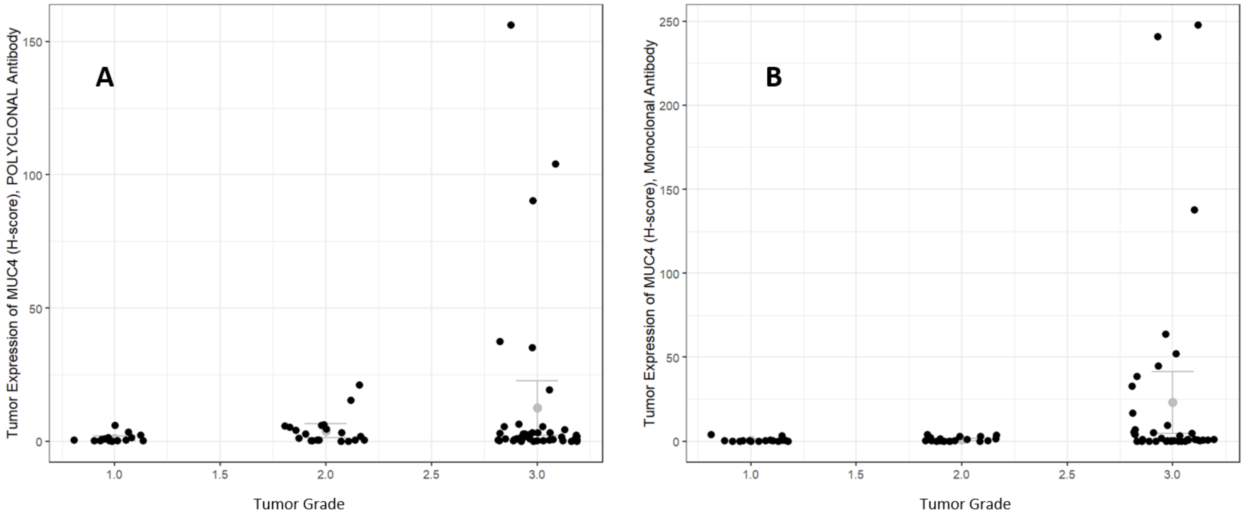

Figure S7. Graph of tissue expression of MUC4 in malignant pancreatic tumors by histologic grade using immunohistochemistry with (A) polyclonal and (B) monoclonal antibodies. Although the majority of tissue samples are negative, tumors that are significantly positive for the expression of MUC4 are only observed in high-grade (grade III) pancreatic ductal adenocarcinomas.

Table S1. Clones that were tested for glycan-specificity.3C7.D8.E11.F5 (blue arrow) was chosen for further studies based on selectivity and affinity.

Screening Preference: Glyco/Nonmod	Cell line list
Both	4H4.D5.D12.E11.C4
Both	4H4.D5.D12.E11.B2
Both	14A1.F3.H1.C12
Both	14A1.F3.H1.A8
Unmodified	20D9.G5.C8.D6.E2.G4
Unmodified	20D9.G5.C8.D6.E2.F8
Glyco	1H12.C8.G4.H10.F10
Glyco	1H12.C8.G4.H10.E2
Unmodified	1B4.B7.G11.F5
Unmodified	1B4.B7.G11.E11
Glyco	3C7.D8.E11.F6
Glyco	3C7.D8.E11.F5

A

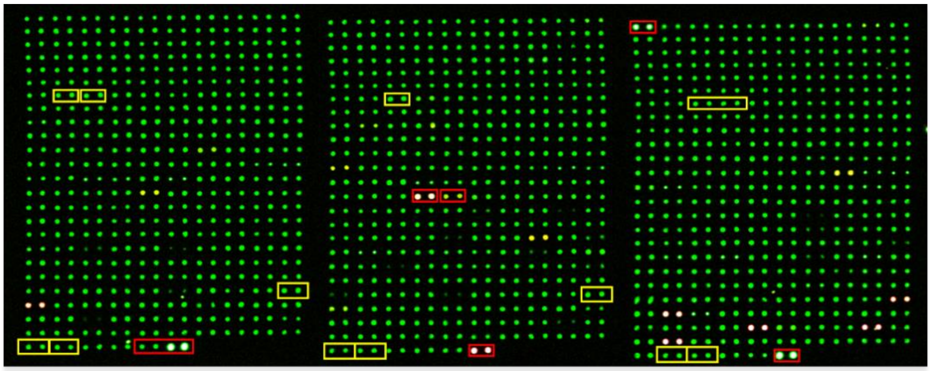

B

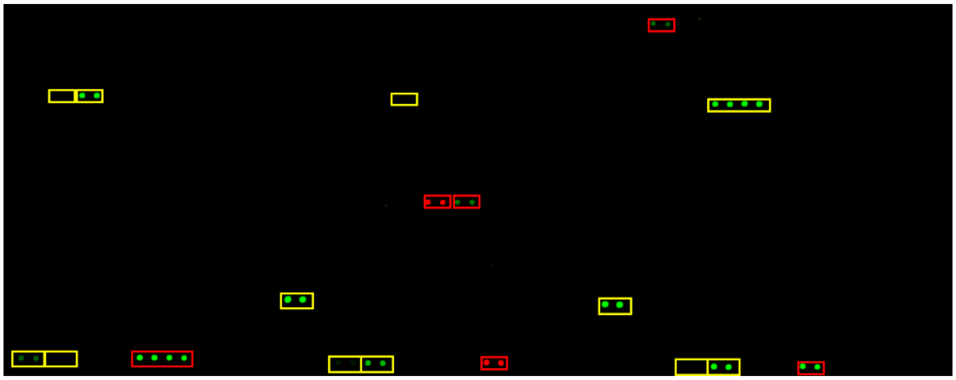

C

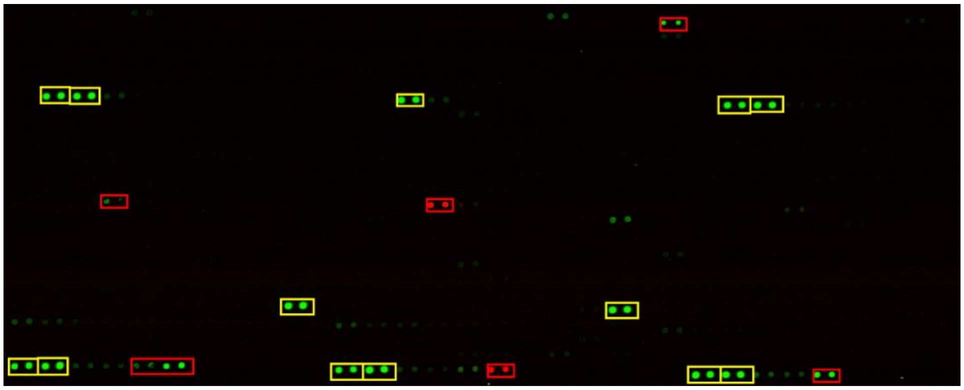

Figure S8: (**A**) Prescan image of the printed slides were taken to evaluate slide quality. Missing or corrupted spots were flagged and excluded from further analysis. (**B** and **C**) Following experimentation, slides were scanned to evaluate binding of mAb (**B**) and pAb (**C**) on the glycan array. Spots within yellow boxes represent the Muc4-peptides/Muc4-Tf-peptide neoglycoprotein components on the array. Spots within red boxes are control spots, such as mouse IgG, Rabbit IgG, and Cy3.

BSA Conjugates. The general procedure for BSA conjugations was the following: To 6.7 mg of BSA dissolved in 1 mL of conjugation buffer (1x PBS, EDTA 1 mM, pH 7.3) was added sulfo-GMBS (100 equivalents) dissolved in 100 µL of conjugation buffer and the mixture was stirred for 2 h at 0°C. Excess sulfo-GMBS was removed by filtration using appropriate molecular weight cutoff (spin)filters, by centrifugation at 5000 g followed by washing with water (3x). Concomitantly, any disulfide species contaminating the thiol substrates were reduced with TCEP resin (Sigma-Aldrich) according to the manufacturer's protocol. This freshly-reduced thiol substrate was reacted with the GMBS-BSA for 18 h and the excess buffer was removed by a similar filtration method to that described above. The BSA conjugates were evaluated by Matrix Assisted Laser Desorption Ionization-Time of Flight (MALDI TOF) mass spectrometry (below).

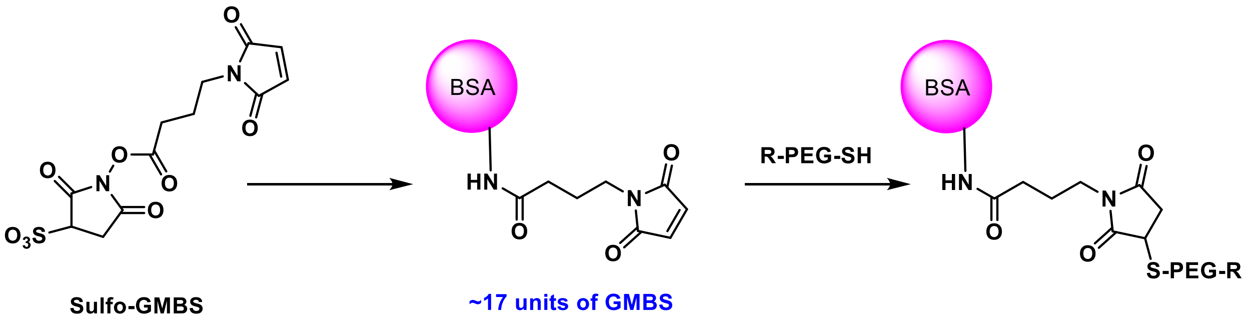

Scheme S1. General synthesis of BSA conjugates

Table S2. Molecular weights of the BSA conjugates and calculation of ligand density.

R group	Mass of R group	Mass of Malemide-BSA	Mass of Conjugate-BSA	Units Conjugated
Peg-SH	554.29	67330.73	76127.32	~16
TSSASTGHATPLPVTD-Peg-SH	2077.33	67330.73	86510.07	~9
TSSA-(TF)S-TGHATPLPVTD-Peg-SH	2441.16	67330.73	88381.76	~8
TSSASTGH-Peg-SH	1281.61	67330.73	83035.1	~9
TSSA-(TF)S-TGH-Peg-SH	1647.76	67330.73	84299.84	~10
ATPLPVTD-Peg-SH	1348.62	67330.73	84747.71	~13

MALDI-TOF Mass Spectrometry of BSA Conjugates. MALDI mass spectra were collected on a Shimadzu Axima Confidence MALDI-TOF mass spectrometer equipped with a high mass CovalX HM4 detector operated in linear positive ion mode. The samples were prepared for MALDI analysis by desalting the BSA conjugates (1.0 mg/mL) using a 0.5 mL 30K Amicon Ultra centrifugal filter. Samples were spotted on an Axima 384 well sample plate using the overlayer method with sinapinic acid as the matrix.

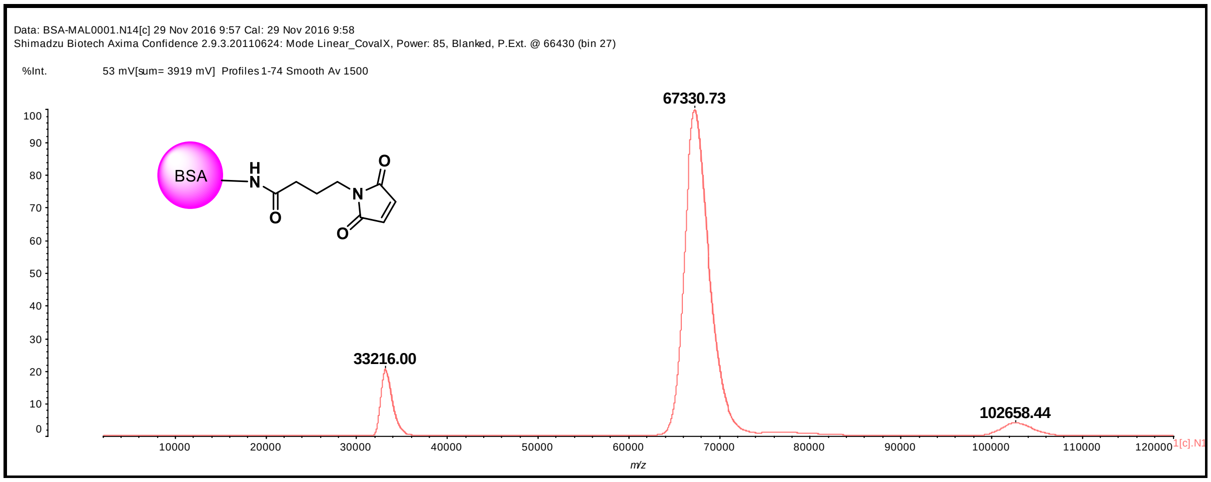

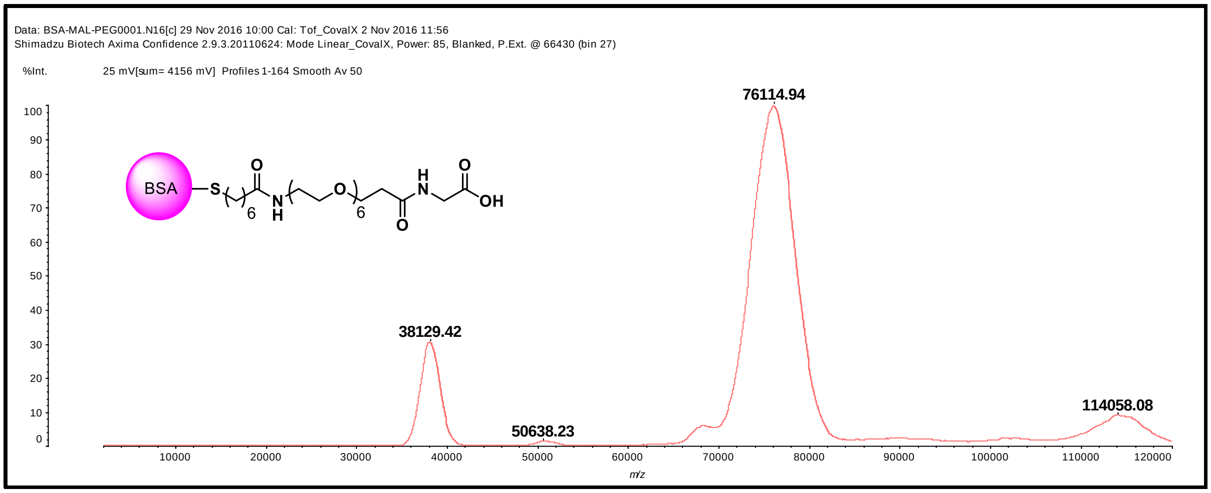

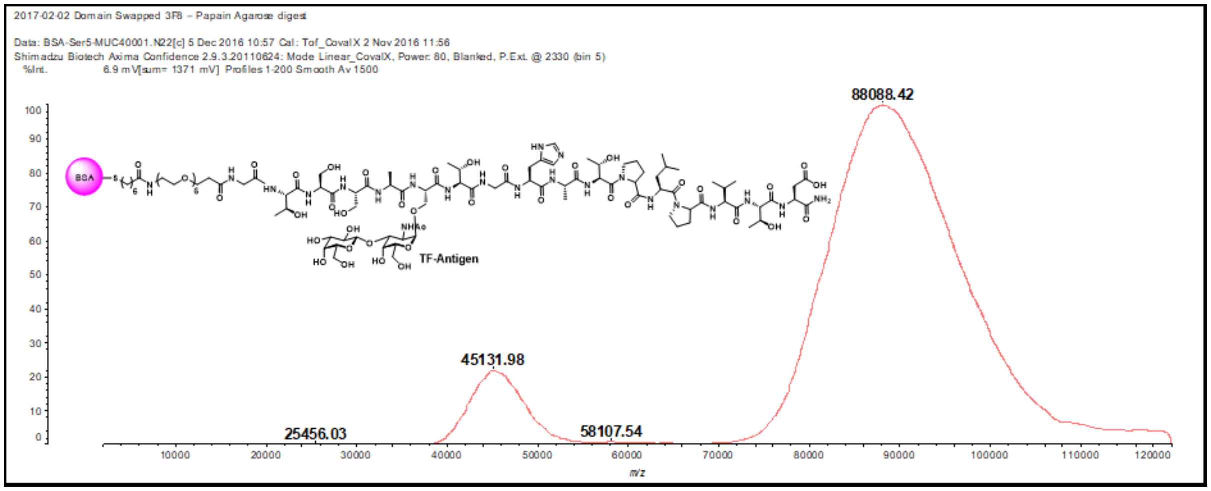
2017-02-02 Domain Swapped 3F8 – Papain Agarose digest
Data: BSA-Ser5-MUC40001.N22[c] 5 Dec 2016 10:57 Cal: Tof_CovalX 2 Nov 2016 11:56
Shimadzu Biotech Axima Confidence 2.9.3.20110624: Mode Linear_CovalX, Power: 80, Blanked, P.Ext. @ 2330 (bin 5)
%Int.
6.9 mV[sum= 1371 mV] Profiles 1-200 Smooth Av 1500
88088.42
45131.98
25456.03
58107.54
BSA
TF-Antigen
m/z

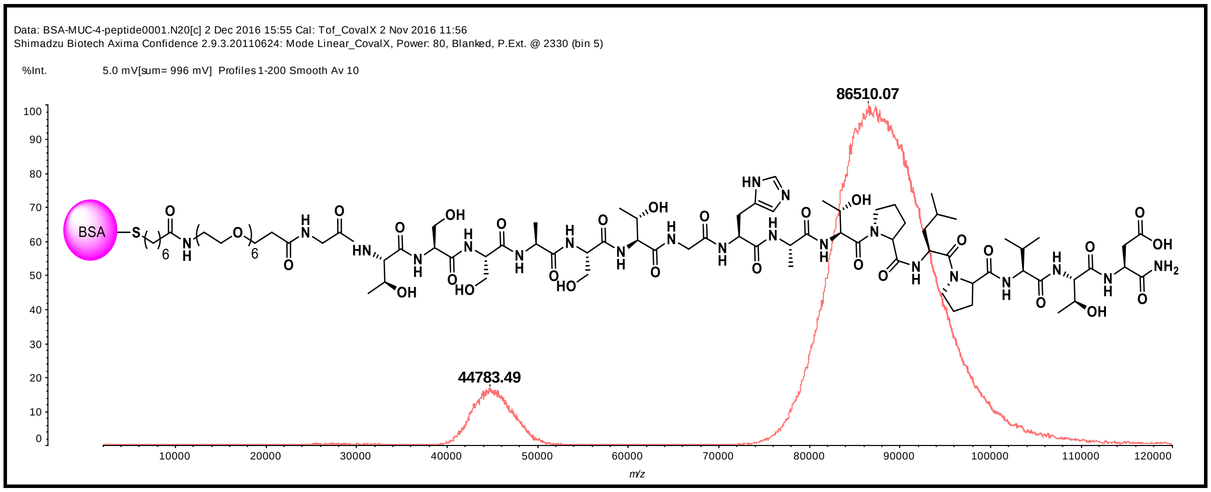
Data: BSA-MUC-4-peptide0001.N20[c] 2 Dec 2016 15:55 Cal: Tof_CovalX 2 Nov 2016 11:56
Shimadzu Biotech Axima Confidence 2.9.3.20110624: Mode Linear_CovalX, Power: 80, Blanked, P.Ext. @ 2330 (bin 5)
%Int.
5.0 mV[sum= 996 mV] Profiles 1-200 Smooth Av 10
86510.07
44783.49
BSA
m/z

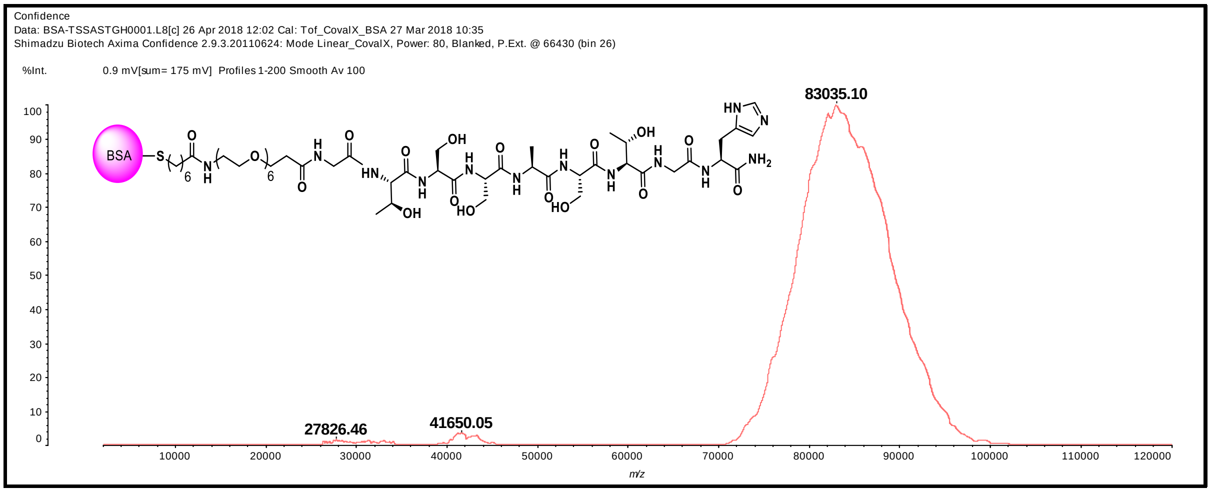
Confidence
Data: BSA-TSSASTGH0001.L8[c] 26 Apr 2018 12:02 Cal: Tof_CovalX_BSA 27 Mar 2018 10:35
Shimadzu Biotech Axima Confidence 2.9.3.20110624: Mode Linear_CovalX, Power: 80, Blanked, P.Ext. @ 66430 (bin 26)
%Int.
0.9 mV[sum= 175 mV] Profiles 1-200 Smooth Av 100
83035.10
27826.46
41650.05
BSA
m/z

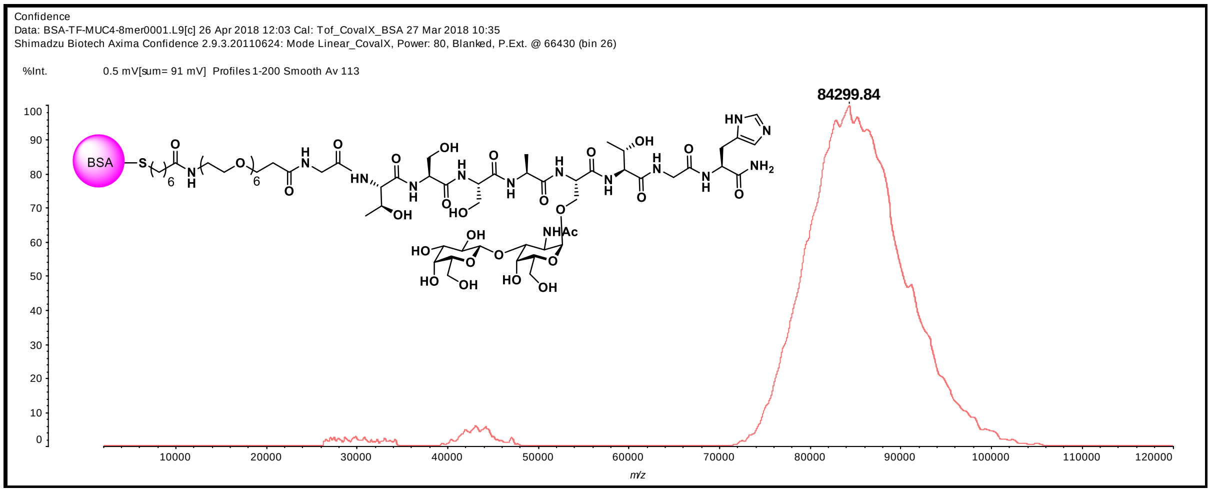
Confidence
Data: BSA-TF-MUC4-8mer0001.L9[c] 26 Apr 2018 12:03 Cal: Tof_CovalX_BSA 27 Mar 2018 10:35
Shimadzu Biotech Axima Confidence 2.9.3.20110624: Mode Linear_CovalX, Power: 80, Blanked, P.Ext. @ 66430 (bin 26)
%Int.
0.5 mV[sum= 91 mV] Profiles 1-200 Smooth Av 113
84299.84
BSA
m/z

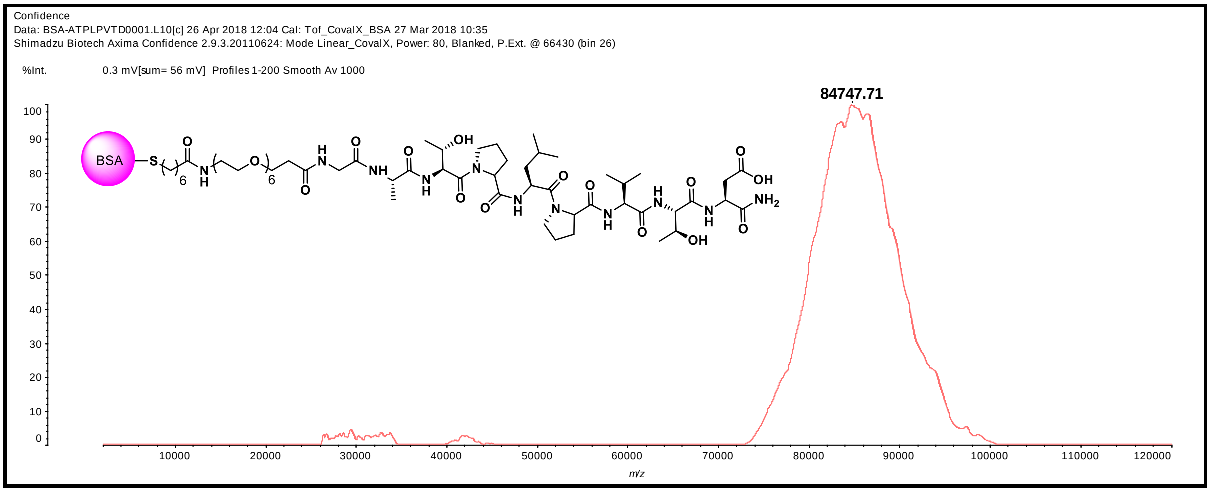
Confidence
Data: BSA-ATPLPVTD0001.L10[c] 26 Apr 2018 12:04 Cal: Tof_CovalX_BSA 27 Mar 2018 10:35
Shimadzu Biotech Axima Confidence 2.9.3.20110624: Mode Linear_CovalX, Power: 80, Blanked, P.Ext. @ 66430 (bin 26)
%Int.
0.3 mV[sum= 56 mV] Profiles 1-200 Smooth Av 1000
84747.71
BSA
m/z
